# Lymphoplasmapheresis versus plasma exchange in severe myasthenia gravis: a retrospective cohort study

**DOI:** 10.3389/fneur.2023.1212868

**Published:** 2023-06-27

**Authors:** Weiwei Duan, Fei Jiang, Haobing Cai, Bijuan Li, Song Ouyang, Weifan Yin, Qiuming Zeng, Huan Yang

**Affiliations:** ^1^Department of Neurology, Xiangya Hospital, Central South University, Changsha, Hunan, China; ^2^Department of Blood Transfusion, Xiangya Hospital, Central South University, Changsha, Hunan, China; ^3^Department of Neurology, The Affiliated Changsha Hospital of Xiangya School of Medicine, Central South University, Changsha, Hunan, China; ^4^The “Double-First Class” Application Characteristic Discipline of Hunan Province (Clinical Medicine), Changsha Medical University, Changsha, China; ^5^Department of Neurology, The Second Xiangya Hospital, Central South University, Changsha, Hunan, China

**Keywords:** myasthenia gravis, lymphoplasmapheresis, plasma exchange, efficacy, therapy

## Abstract

**Background:**

Lymphoplasmapheresis (LPE) is a new therapy developed on the basis of traditional plasma exchange (PE) in combination with leukapheresis. Currently, it remains unclear whether PE and LPE show differences in efficacy for severe MG.

**Methods:**

A retrospective analysis was conducted on 198 MG patients, 75 in the PE group and 123 in the LPE group, and the patients’ Myasthenia Gravis Foundation of America (MGFA) Clinical Classification was Class IV. The treatment outcome was the change in Quantitative Myasthenia Gravis Score (QMGS) from baseline to the end of treatment. Propensity score matching (PSM) was applied for the balance of confounders between the two groups.

**Results:**

In this study cohort, the safety profile of LPE and PE was good and no serious adverse events were observed. Based on PSM, 62 patients treated with LPE and 62 patients treated with PE were entered into a comparative efficacy analysis. In the PE group, patients underwent a total of 232 replacements, with a mean of 3.74. PE significantly improved the patients’ QMGS performance, with the mean QMGS decreasing from 22.98 ± 4.03 points at baseline to 18.34 ± 5.03 points after treatment, a decrease of 4.68 ± 4.04 points (*p* < 0.001). A decrease of ≥3 points in QMGS was considered a significant improvement, with a treatment response rate of 67.7% in the PE group. In the LPE group, patients received a total of 117 replacements, with a mean of 1.89. The patients’ mean QMGS was 23.19 ± 4.11 points at baseline and was 16.94 ± 5.78 points after treatment, a decrease of 6.26 ± 4.39 points (*p* < 0.001). The improvement in QMGS was more significant in patients treated with LPE compared to the PE group (*p* = 0.039). The treatment response rate in the LPE group was 79%, which was not significantly different compared to the PE group (*p* = 0.16). The LEP group had a shorter mean length of stay compared to the PE group (10.86 ± 3.96 vs. 12.14 ± 4.14 days), but the difference was not statistically significant (*p* = 0.13). During the 2-month follow-up period, LPE may be associated with better functional outcomes for patients, with lower QMG score and relapse rate. LPE and PE were both effective in reducing the levels of inflammatory cytokines (TNF-α, IL-1β, and IL-6) and AChR-Ab. Compared to PE, LPE was superior in the reduction of AChR-Ab titer.

**Conclusion:**

In severe MG, LPE may be a more preferred treatment option than PE. It achieves treatment outcomes that are not inferior to or even better than PE with fewer replacements. This study provides further evidence to support the application of LPE as a new treatment option for MG.

## 1. Introduction

Myasthenia gravis (MG) is an acquired autoimmune disease involving the postsynaptic membrane of the neuromuscular junction (1). Currently, it is believed that the presence of autoantibodies (Abs) targeting the acetylcholine receptor (AChR), muscle specific kinase (MuSK), and low-density lipoprotein receptor-related protein 4 (LRP4) is the main driving factor for the pathophysiological processes in most patients (2). Additionally, autoantibodies to muscle-related proteins, such as titin-Ab and ryanodine receptor (RyR)-Ab, have been detected in some patients (3). MG is characterized by fluctuating muscle weakness, with ocular muscles typically being the first to be affected (ocular myasthenia gravis, OMG), with diplopia and ptosis (4). Most patients’ condition gradually progresses within a few years, with generalized symptoms (generalized myasthenia gravis, gMG), involving facial, neck, limb, and bulbar muscles symmetrically, resulting in difficulty in raising head, weakness of limbs, dysphagia, and dysarthria (5). In severe cases, respiratory muscle paralysis may occur (myasthenia crisis), which is life-threatening (6).

In patients with severe MG, not only is the ability to perform daily life severely impaired, but life-threatening myasthenia crisis may also occur, greatly increasing the disease burden of patients. Therefore, rapid improvement in symptoms is crucial for patients with severe MG. Currently, plasma exchange (PE) and intravenous immunoglobulin (IVIG) are the first-line treatment options to achieve rapid clinical response in severe MG (7, 8). PE is a therapeutic procedure in which a patient’s plasma is separated and removed from the blood by a medical device and replaced with a colloidal solution (plasma and or albumin) or a combination of colloidal/crystal solution. The effective removal of pathogenic factors (autoantibodies, etc.) from the patient’s plasma during this process is the main mechanism of PE treatment for autoimmune diseases (9). Currently, it is considered that PE may be a better choice compared to IVIG for the therapy of severe MG, with a better efficacy and faster onset of action (10).

Lymphoplasmapheresis (LPE) is a new therapy that combines lymphocyte apheresis and traditional PE. Compared with PE, it removes not only soluble pathological immune factors such as adhesion molecules, complements, cytokines, and autoantibodies from the plasma, but also immunoreactive cells such as sensitized B and T lymphocytes, resulting in more effective and sustained control of pathological immune responses (11). The efficacy of LPE has been demonstrated in several autoimmune diseases, such as steroid-resistant neuromyelitis optica spectrum disorders, Guillain-Barré syndrome, and refractory severe autoimmune skin diseases (11–14). The results of previous study at our center have shown that LPE is a safe and effective treatment for the exacerbation of MG (15), but it is currently unclear whether it is superior to PE. Thus, this study aimed to compare the therapeutic effect of LPE and PE in severe MG, in order to provide further reference for the application of LPE in MG.

## 2. Methods

### 2.1. Data sources

The initial study cohort included 236 patients with severe MG who were treated with PE or LPE from November 2016 to January 2022 at the Department of Neurology, Xiangya Hospital, Xiangya Second Hospital, and Changsha First Hospital. The diagnosis basis of MG was as follows: (1) typical symptoms of muscle weakness (fluctuating and easy fatigability). (2) positive test for MG-related autoantibodies. Electromyography and neostigmine test were applied to assist the diagnosis of antibody-negative cases (6). In this study, the definition of severe MG was that the patient’s Myasthenia Gravis Foundation of America (MGFA) Clinical Classification was Class IV (16). The MGFA Clinical Classification indicates the severity of disease, with Class IV suggesting severe involvement of muscle groups. 38 patients were excluded due to incomplete clinical information. Thus, 198 patients were incorporated into the final study cohort, including 123 patients receiving LPE treatment and 75 patients receiving PE treatment.

The data including demographic characteristics and MG-related clinical information were collected. The data on immunological indexes at baseline and after intervention were also collected (some patients were missing), including AChR-Ab (LPE group, *n* = 47; PE group, *n* = 41), TNF-α (LPE group, *n* = 52; PE group, *n* = 56), IL-6 (LPE group, *n* = 52; PE group, *n* = 56), and IL-1β (LPE group, *n* = 52; PE group, *n* = 56). The time points for the collection of immunological indicators were within 3 days before and after LPE or PE treatment. The reports of treatment-related adverse events were collected for clinical safety assessment. In addition, data were collected for the follow-up period, including Quantitative Myasthenia Gravis (QMG) scores and relapses. In this study, relapse was defined as the patient’s re-hospitalization due to the deterioration of MG. The Ethics Committees of Xiangya Hospital, Xiangya Second Hospital, and Changsha First Hospital approved this study protocol.

### 2.2. LPE and PE procedure

LPE was performed as previously described (12, 13, 17). In each procedure, about 0.7–0.8 plasma volume (20–25 ml/kg) was replaced by the fresh frozen plasma with an equal volume, and 2–3 × 10^9 lymphocytes were removed. The LPE regimen was 1 procedure conducted every 3 days, with 1–3 procedures in total. PE was performed according to the standard procedures (18, 19), with a replacement of approximately 1–1.5 times plasma volume (35–40 ml/kg) each procedure. The PE course consisted of 3–5 procedures, once every other day.

### 2.3. Synchronous therapy regimen

During the treatment of LPE or PE, long-term immunosuppressive maintenance therapy with corticosteroids and/or non-steroidal immunosuppressants was administered simultaneously (Table 1). The initial dose of corticosteroids (prednisone) was 20 mg/day, increasing by 5 mg every 3 days to 1 mg/kg/day for maintenance. It was permissible to use acetylcholinesterase inhibitors (AChEIs), such as pyridostigmine bromide tablet. A high-dose corticosteroid pulse was not given to any of the patients during either the LPE or PE phases of treatment. Additionally, patients with co-infections received concomitant treatment with sensitive antibiotics.

**Table 1:** Baseline characteristics of patients in PE and LPE groups before propensity score matching.

Characteristics	PE (*n* = 75)	LPE (*n* = 123)	*p* value
Gender (female) (*n*, %)	54 (72.0%)	76 (61.8%)	0.14
Age (years, mean ± SD)	46.63 ± 15.95	45.24 ± 16.12	0.56
Disease duration (month, mean ± SD)	48.92 ± 68.50	46.64 ± 71.28	0.76
Thymoma (*n*, %)	17 (22.7%)	25 (20.3%)	0.70
Thymic hyperplasia (*n*, %)	3 (4.0%)	4 (3.3%)	0.78
History of thymectomy (*n*, %)	17 (22.7%)	28 (22.8%)	0.98
Other autoimmune diseases (*n*, %)	15 (20.0%)	24 (19.5%)	0.93
Co-infection (*n*, %)	22 (29.3%)	30 (24.4%)	0.44
Immunotherapy before treatment (*n*, %)	42 (56.0%)	66 (53.7%)	0.75
History of myasthenic crisis (*n*, %)	8 (10.7%)	14 (11.4%)	0.88
MGFA IVb (*n*, %)	63 (84.0%)	101 (82.1%)	0.73
Baseline QMGS (mean ± SD)	23.03 ± 4.03	23.40 ± 4.25	0.54
AChR-Ab (*n*, %)	69 (92.0%)	110 (89.4%)	0.55
MuSK-Ab (*n*, %)	1 (1.3%)	4 (3.3%)	0.40
Titin-Ab (*n*, %)	20 (26.7%)	26 (21.1%)	0.37
RyR-Ab (*n*, %)	10 (13.3%)	15 (12.2%)	0.82
Simultaneous oral immune drugs (*n*, %)			
Prednisone monotherapy	18 (24.0%)	9 (7.3%)	0.001**
Prednisone and tacrolimus	41 (54.7%)	88 (71.5%)	0.016*
Prednisone and mycophenolate mofetil	5 (6.7%)	10 (8.1%)	0.71
Prednisone and azathioprine	3 (4.0%)	5 (4.1%)	0.98
Tacrolimus monotherapy	5 (6.7%)	8 (6.5%)	0.96
Mycophenolate mofetil monotherapy	2 (2.7%)	3 (2.4%)	0.92
Length of stay	11.85 ± 4.27	11.26 ± 4.19	0.34

*p < 0.05, **p < 0.01.

### 2.4. Efficacy evaluation

In the evaluation of therapeutic effect, the treatment outcome was the change in QMGS from baseline to the end of treatment. The QMGS is designed to quantify the severity of disease and consists of 13 items to assess the function of the ocular, bulbar, limb, and respiratory muscles. Each item has a possible score from 0 to 3, with a total possible score of 39, with higher scores indicating more severe impairment (20). The threshold for determining treatment effectiveness was that QMGS was reduced by 3 points or more (21). AChEIs were discontinued 6–8 h prior to each scoring, and the time points for scoring were within 3 days prior to the initiation of PE or LPE and within 3 days after completion. For patients who received multiple scores, the baseline score and the last score were used for efficacy analysis.

### 2.5. Statistical analysis

Statistical analysis of this study was performed using SPSS 26.0 software (IBM, USA). Categorical variables were expressed as counts and percentages. Continuous variables were expressed as mean ± standard deviation (SD). Paired t-test or Wilcoxon signed-rank test were applied for the comparison of immune indicators and QMGS before and after intervention. Two-sample t-test or Mann–Whitney U test was used for the comparison of quantitative data between the two groups, and chi-square test or Fisher exact test was used for the comparison of qualitative data. Propensity score matching (PSM) was used to equalize the confounders between the two groups with a matching ratio of 1:1 and a matching tolerance of 0.03. In this study, *p* < 0.05 was used as the cut-off value for statistically significant differences.

## 3. Results

### 3.1. Baseline characteristics

**Figure 1:**
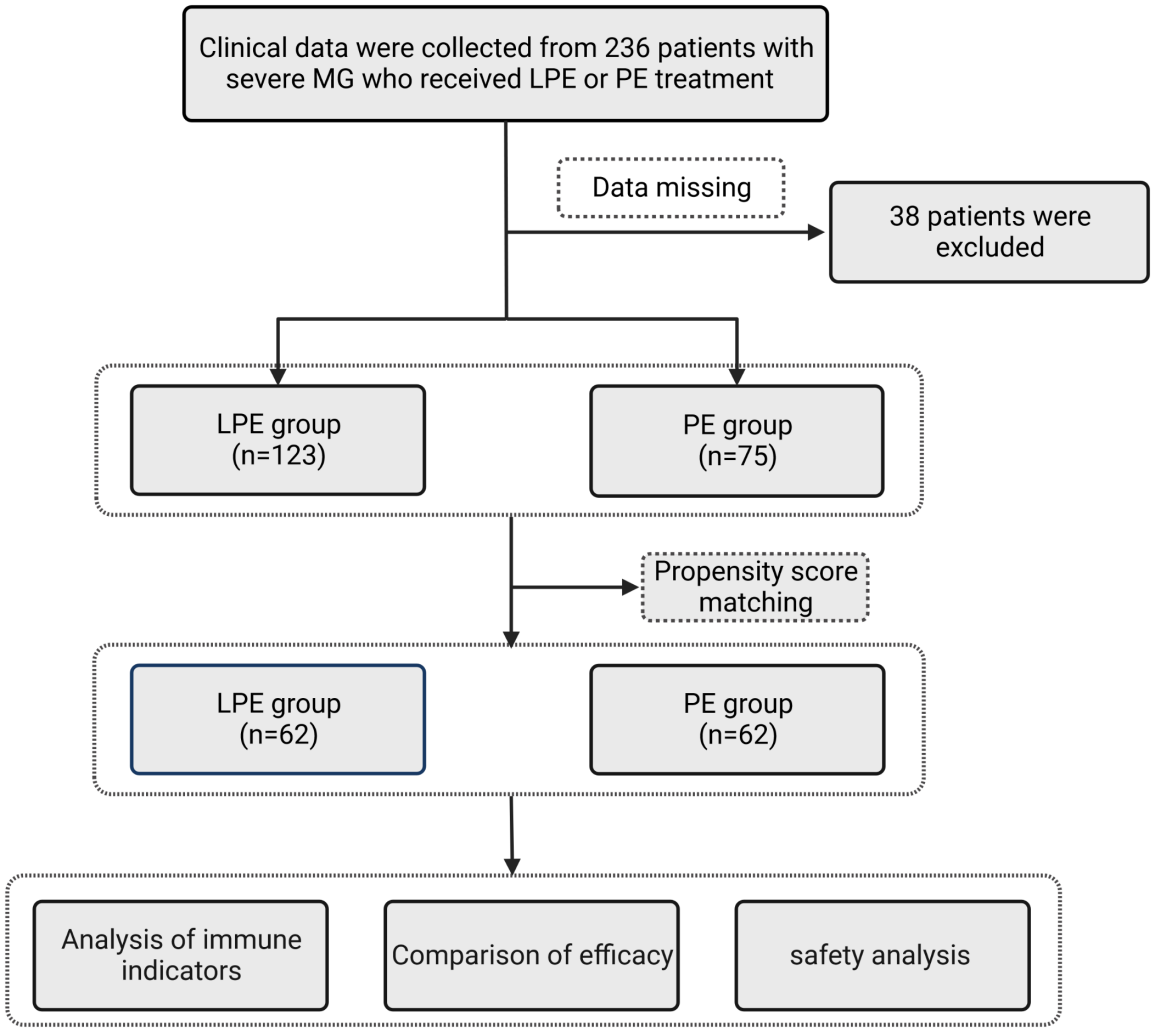
The flowchart designed for this study.

Figure 1 demonstrates the workflow of this study. The clinical features of the included patients are summarized in Table 1. Both the PE and LPE groups were predominantly female, accounting for 72.0% and 61.8%, respectively. The mean age of LPE group was 45.24 ± 16.12 years old, and that of PE group was 46.63 ± 15.95 years old. 20.3% (25 cases) of patients in the LPE group were accompanied by thymoma, and the proportion in the PE group was 22.7% (17 cases). The proportion of patients with a history of thymectomy was 22.8% (28 cases) in the LPE group and 22.7% (17 cases) in the PE group. The baseline QMGS of LPE group was 23.40 ± 4.25 points, and that of PE group was 23.03 ± 4.03 points. Regarding the autoantibodies related to MG, the AChR-Ab was identified in the majority of patients in both groups, accounting for 89.4% (LPE group) and 92% (PE group), respectively. On a synchronous oral immunotherapy regimen, more patients in the PE group received prednisone monotherapy compared with the LPE group (24.0% vs. 7.3%, *p* = 0.001). In the LPE group, more patients were treated with a combination of prednisone and tacrolimus (71.5% vs. 54.7%, *p* = 0.016), which was considered to be related to clinical medication preferences in different centers. In addition, the mean length of hospital stay was 11.85 ± 4.27 days for patients in the PE group and 11.26 ± 4.19 days for patients in the LEP group, with no significant difference (*p* = 0.34).

### 3.2. Comparison of the efficacy between PE and LPE

**Figure 2:**
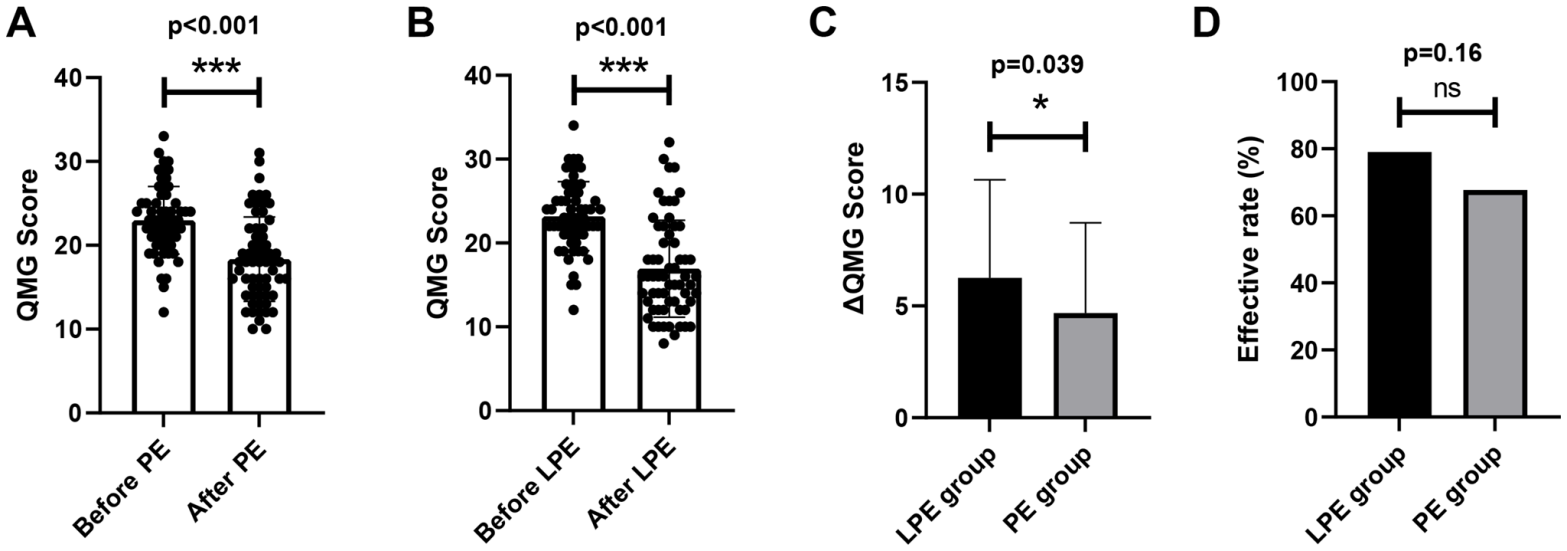
Comparison of the efficacy of LPE and PE. **(A)** Changes in QMGS before and after treatment in the PE group (*n* = 62). **(B)** Changes in QMGS before and after treatment in the LPE group (*n* = 62). **(C)** Comparison of QMGS improvement between the LPE and PE groups. **(D)** Comparison of effective rate between the LPE and PE groups. The bar represents the mean value; the error bar represents the standard deviation. ***p < 0.001; *p < 0.05; ns, no significance.

**Figure 3:**
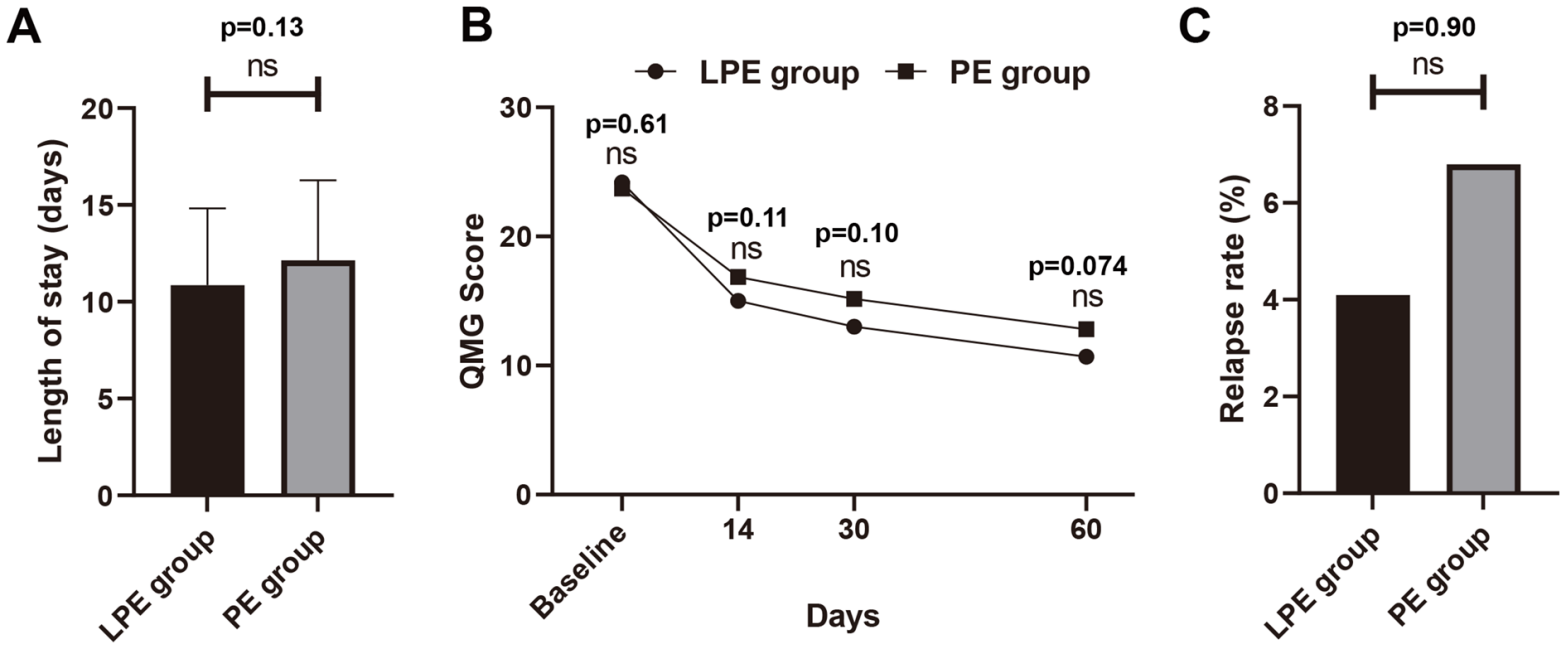
Comparison of LPE and PE in the follow-up period. **(A)** Comparison of hospital stay between the LPE group (*n* = 62) and PE group (*n* = 62). **(B)** Comparison of QMG scores between the LPE group (*n* = 49) and the PE group (*n* = 44) during follow-up. **(C)** Comparison of relapse rates between the LPE group and PE group. The bar represents the mean value; the error bar represents the standard deviation; ns, no significance.

We further compared the efficacy of LPE and PE. In order to eliminate the interference of potential confounding factors on the analysis, we first performed the propensity score matching between the two groups, and 62 pairs of patients were successfully matched. No significant differences in baseline characteristics were identified between the two groups after matching (Table 2, *p* > 0.05). In the PE group, patients received a total of 232 replacements with an average of 3.74; the baseline QMG score was 22.98 ± 4.03 points, and the QMG score after PE was 18.34 ± 5.03 points, a decrease of 4.68 ± 4.04 points, with statistical significance (Figure 2A, *p* < 0.001). In the LPE group, patients received a total of 117 replacements with an average of 1.89; the mean QMG score was 23.19 ± 4.11 points at baseline and was 16.94 ± 5.78 points after LPE, which decreased by 6.26 ± 4.39 points and improved obviously (Figure 2B, *p* < 0.001). Compared with the PE group, patients receiving LPE treatment showed more significant improvement in QMG score (6.26 ± 4.39 vs. 4.68 ± 4.04 points, *p* = 0.039, Figure 2C). A decline in QMG score ≥ 3 points was regarded as a significant improvement, with a treatment effectiveness rate of 67.7% (42/62) in the PE group and 79.0% (49/62) in the LPE group, with no significant difference between them (Figure 2D, *p* = 0.16). Moreover, the mean length of hospital stay in the LEP group was 10.86 ± 3.96 days, which was shorter than that in the PE group (12.14 ± 4.14 days), but the difference was not statistically significant (*p* = 0.13, Figure 3A). The above results suggest that PE and LPE both can effectively improve the symptoms of patients, but LPE performed better.

**Table 2:** Baseline characteristics of patients in PE and LPE groups after propensity score matching.

Characteristics	PE (*n* = 62)	LPE (*n* = 62)	*p* value
Gender (female) (*n*, %)	44 (71.0%)	40 (64.5%)	0.44
Age (years, mean ± SD)	47.18 ± 16.09	47.79 ± 16.27	0.83
Disease duration (month, mean ± SD)	53.19 ± 72.73	51.19 ± 75.75	0.88
Thymoma (*n*, %)	11 (47.8%)	12 (52.2%)	0.82
Thymic hyperplasia (*n*, %)	3 (4.8%)	1 (1.6%)	0.62
History of thymectomy (*n*, %)	17 (27.4%)	15 (24.2%)	0.68
Other autoimmune diseases (*n*, %)	13 (21.0%)	12 (19.4%)	0.82
Co-infection (*n*, %)	15 (24.2%)	18 (29.0%)	0.54
Immunotherapy before treatment (*n*, %)	34 (54.8%)	34 (54.8%)	1.00
History of myasthenic crisis (*n*, %)	6 (9.7%)	6 (9.7%)	1.00
MGFA IVb (*n*, %)	51 (82.3%)	50 (80.6%)	0.82
Baseline QMGS (mean ± SD)	22.98 ± 4.03	23.19 ± 4.11	0.78
AChR-Ab (*n*, %)	57 (91.9%)	59 (95.2%)	0.72
MuSK-Ab (*n*, %)	1 (1.6%)	0 (0.0%)	1.00
Titin-Ab (*n*, %)	17 (27.4%)	18 (29.0%)	0.84
RyR-Ab (*n*, %)	9 (14.5%)	7 (11.3%)	0.59
Simultaneous oral immune drugs (*n*, %)			
Prednisone monotherapy	7 (11.3%)	8 (12.9%)	0.78
Prednisone and tacrolimus	41 (66.1%)	43 (69.4%)	0.70
Prednisone and mycophenolate mofetil	4 (6.5%)	3 (4.8%)	0.69
Prednisone and azathioprine	3 (4.8%)	3 (4.8%)	1.00
Tacrolimus monotherapy	5 (8.1%)	4 (6.5%)	0.73
Mycophenolate mofetil monotherapy	2 (3.2%)	1 (1.6%)	0.56

Furthermore, we further analyzed changes in patients’ QMG scores from baseline to 14, 30, and 60 days post-treatment. Due to the lack of complete follow-up data, some patients were excluded from this analysis, with 13 excluded from the LPE group and 49 patients included, and 18 excluded from the PE group and 44 patients included. The results indicated that the QMG score of patients after LPE or PE treatment showed a gradual decline trend (Figure 3B). No significant difference in baseline QMG score was identified between the LPE and PE groups (24.18 ± 4.56 vs. 23.71 ± 4.23 points, *p* = 0.61). At each follow-up time point, the mean QMG score of patients treated with LPE was lower than that of patients treated with PE (at 14 days, 15.02 ± 5.61 vs. 16.86 ± 5.37 points, *p* = 0.11; at 30 days, 13.00 ± 6.63 vs. 15.16 ± 6.10 points, *p* = 0.10; at 60 days, 10.68 ± 5.84 vs. 12.80 ± 5.43 points, *p* = 0.074), but no statistically significant differences were found (Figure 3B). In addition, the relapse rate of patients receiving LPE treatment was 4.1% (2/49) and that of patients receiving PE treatment was 6.8% (3/44), with no significant difference (*p* = 0.90, Figure 3C). These results suggest that LPE may be associated with better functional outcomes in patients during follow-up.

### 3.3. Effects of LPE and PE on inflammatory factors and autoantibody

**Figure 4:**
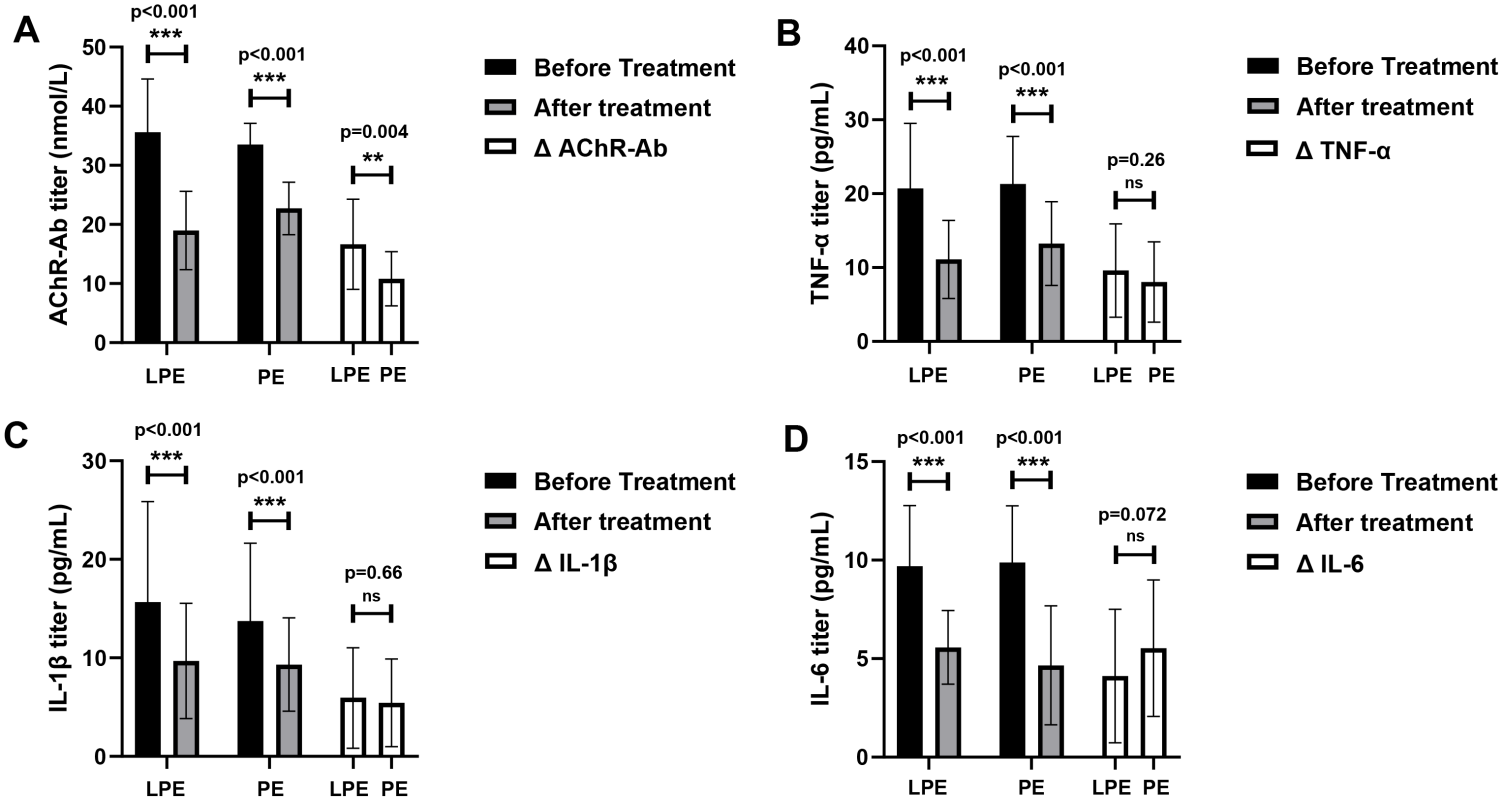
Changes of immune indicators before and after LPE and PE treatment. **(A)** Changes of AChR-Ab titer before and after treatment; comparison of the change magnitude in AChR-Ab titer between the LPE and PE groups. **(B)** Changes of TNF-α level before and after treatment; comparison of the change magnitude in TNF-α between the LPE and PE groups. **(C)** Changes of IL-1β level before and after treatment; comparison of the change magnitude in IL-1β between the LPE and PE groups. **(D)** Changes of IL-6 level before and after treatment; comparison of the change magnitude in IL-6 between the LPE and PE groups. The bar represents the mean value; the error bar represents the standard deviation. ***p < 0.001, **p < 0.01; ns, no significance.

We further assessed the effects of LPE and PE on immune-related laboratory indexes. The levels of AChR-Ab (35.60 ± 8.99 vs. 18.97 ± 6.63 nmol/L, *p* < 0.001, Figure 4A), TNF-α (20.70 ± 8.81 vs. 11.10 ± 5.30 pg/ml, *p* < 0.001, Figure 4B), IL-1β (15.67 ± 10.18 vs. 9.70 ± 5.85 pg/ml, *p* < 0.001, Figure 4C), and IL-6 (9.69 ± 3.08 vs. 5.57 ± 1.87 pg/ml, *p* < 0.001, Figure 4D) decreased significantly after LPE treatment. After PE treatment, the levels of AChR-Ab (33.52 ± 3.60 vs. 22.72 ± 4.44 nmol/L, *p* < 0.001, Figure 4A), TNF-α (21.33 ± 6.42 vs. 13.26 ± 5.67 pg/ml, *p* < 0.001, Figure 4B), IL-1β (13.75 ± 7.89 vs. 8.32 ± 4.73 pg/ml, *p* < 0.001, Figure 4C), and IL-6 (9.88 ± 2.87 vs. 4.66 ± 3.02 pg/ml, *p* < 0.001, Figure 4D) were also significantly decreased. No significant differences were identified in the magnitude of IL-1β, TNF-α, and IL-6 decline between the two groups (Figures 4B–D, *p* > 0.05). Compared with PE, LPE had a better performance in reducing AChR-Ab titer (16.63 ± 7.63 vs. 10.80 ± 4.59 nmol/L, *p* = 0.004, Figure 4A).

### 3.4. Safety profile of LPE versus PE

In this study cohort, both LPE and PE therapy were well tolerated. Among the 223 replacements of 123 patients in the LPE group, the treatment-related adverse events included allergy (14), citrate reaction (5), hypotension (2), chills (4), and nausea and vomiting (3). Among the 279 replacements of 75 patients in the PE group, the adverse reactions associated with treatment included allergy (16), citrate reaction (6), headache (2), chills (3), vasovagal reaction (1), hypotension (1), hypertension (2), and local bleeding (2). The above adverse reactions were effectively alleviated after symptomatic treatment. No serious adverse events causing death in patients were observed.

## 4. Discussion

MG is an autoimmune disease in which autoantibodies attack postsynaptic membrane-related proteins of neuromuscular junction, resulting in neuromuscular impulse conduction disorder. Fluctuating muscle weakness is its main clinical feature. In severe cases, the ability to daily living is severely impaired due to severe skeletal muscle weakness, and respiratory failure (myasthenic crisis) caused by severe respiratory muscle weakness may be life-threatening. PE is the current first-line therapy for patients with severe MG, which can quickly and effectively relieve symptoms, and its mechanism is to remove the plasma containing large amounts of pathogenic factors during replacement. In a randomized trial evaluating the therapeutic effect of PE in moderate to severe MG, patients had a mean QMGS improvement of 4.7 ± 4.9 points after treatment, and the symptoms were effectively relieved (the improvement of QMGS ≥3 points) in 57% of patients (22). In another randomized trial for MG exacerbation, the remission rate of patients’ symptoms was 66% after PE treatment (23). The above results are similar to those of this study, with 67.7% of patients achieving effective symptom relief and a mean QMG score decrease of 4.68 ± 4.04 points after PE treatment.

LPE is a new treatment based on traditional PE combined with leukapheresis. There is currently no study comparing the therapeutic effect of PE and LPE in severe MG. In the LPE cohort of this study, the mean QMG score was reduced by 6.26 ± 4.39 points after treatment, which was significantly better than that of patients receiving PE treatment. In addition, the clinical effective rate of the LPE group was 75.6%, which was higher than that of the PE group (66.7%). Although no statistically significant difference was identified between them, it may be associated with the relatively insufficient sample size in this study. It should also be noted that compared with the mean of 3.74 replacements in the PE group, the patients in the LPE group received significantly fewer replacements (mean of 1.89). This not only lowers the possibility of serious adverse events that occur during repeated replacement, greatly cuts down the treatment burden of patients, but also reduces the plasma consumption in the current shortage environment of clinical blood resources, which has important clinical practice implications. In addition, both LPE and PE performed similarly in terms of safety and were well tolerated, with adverse reactions being common with citrate reactions and allergic reactions, which is in accord with the results of previous researches (14, 17).

Previous studies have shown that the efficacy of replacement therapy lasts for about 1–2 months (24, 25). In this study, we also found that the QMG score in both the LPE and PE groups showed a gradual decrease during the 2-month follow-up period. However, it should be noted that patients received a long-term immunosuppressive maintenance therapy at the same time, and although these drugs mostly showed benefit only weeks or months later (7, 25, 26) and had a very limited impact on the short-term efficacy assessment of LPE and PE, it is difficult to determine whether they interfered with our evaluation of durative efficacy of LPE and PE during the follow-up period. In addition, we also found that LPE treatment may be associated with better functional outcomes at 2-month follow-up; the LPE group had a lower mean QMG score at each follow-up time points and a lower relapse rate than the PE group, although no statistically significant differences were identified between them, but this result may be limited by our limited sample size.

In terms of impact on immune-related factors, LPE and PE could effectively reduce the levels of inflammatory cytokines (TNF-α, IL-1β, and IL-6) and AChR-Ab. AChR-Ab is the primary autoantibody responsible for the pathogenesis of MG, and the decrease of its titer is associated with the improvement of clinical symptoms (27). IL-1β induces the differentiation of T helper 17 (Th17) cells (28), which promote inflammatory responses and mediate tissue damage, and participate in MG autoimmunity by regulating B-cell tolerance and the production of AChR-Ab (29). The serum level of IL-1β is significantly elevated in MG patients and correlates with symptoms (30, 31). IL-6 is a pleiotropic inflammatory cytokine that promotes B-cell differentiation and proliferation, induces B-cell maturation into antibody-producing plasma cells, and regulates the immune homeostasis between Tregs and Th17 cells. Its overexpression is involved in the pathogenesis of various inflammatory autoimmune diseases, including MG (32). TNF-α, as a pro-inflammatory cytokine, has the ability to induce a broad secondary inflammatory cascade response (33). In MG, its overexpression affects the immune response by driving the Th1 response, upregulating B cell proliferation and differentiation, and inducing IL-6 production (33, 34). Compared with PE, LPE could reduce the titer of autoantibody in MG patients more effectively. In terms of inflammatory cytokine clearance, LPE obtained similar effect to traditional PE with fewer replacements. We speculate that the better performance of LPE in eliminating immunopathogenic factors is related to its unique advantages in the mechanism of action. The clearance of autoantibodies, inflammatory factors, and other pathological substances in plasma by traditional PE may relieve the feedback inhibition on immunocompetent cells, leading to a rebound in the level of immunopathological substances (17). LPE can not only eliminate soluble pathogenic factors in plasma, but also remove sensitized immunocompetent cells, thus inhibiting the continuous production of pathological factors (11, 14, 17).

In general, this study is the first to compare the efficacy of PE and LPE in MG. Our results suggest that LPE achieves the clinical efficacy not inferior to or even better than PE with fewer replacements in severe MG. The findings of this study provide further evidence to support the application of LPE in MG. Our study also has some limitations. First, selection bias was difficult to avoid due to the limitations of the retrospective design of this study. Second, due to limitations in data availability (e.g., missing data for scores such as ADL, MGC, and MG-QOL15r), we were unable to comprehensively evaluate the efficacy of LPE and PE using multiple scoring systems combined. Furthermore, this study lacked mechanistic exploration. Finally, the sample size of this study was relatively limited, which may also lead to bias in the results. In the future, the results of this study need to be confirmed by more rigorous randomized controlled trials, and the underlying therapeutic mechanism of LPE in severe MG needs to be delved in depth to further strengthen the evidence supporting the application of LPE in the management of severe MG.

## 5. Conclusion

In conclusion, LPE may be a better treatment option than PE for patients with severe MG, offering advantages in terms of efficacy while requiring fewer replacements.

## Data availability statement

The original contributions presented in the study are included in the article/supplementary material, further inquiries can be directed to the corresponding author/s.

## Ethics statement

The studies involving human participants were reviewed and approved by the Ethics Committees of Xiangya Hospital, Xiangya Second Hospital, and Changsha First Hospital. Written informed consent for participation was not required for this study in accordance with the national legislation and the institutional requirements.

## Author contributions

HY and QZ conceived and designed the research. WD, FJ, and HC collected the clinical data. WD analyzed the data. BL participated in research design and data analysis. WD, SO, and WY drafted the manuscript. All authors contributed to the article and approved the submitted version.

## Funding

This research was supported by the National Natural Science Foundation of China (81771364 and 82171399), Science and Technology Innovation Guidance Project of Hunan Province (2020SK53009), Changsha Municipal Natural Science Foundation (kq2007037), and Natural Science Foundation of Hunan Province (2022JJ40724).

## Conflict of interest

The authors declare that the research was conducted in the absence of any commercial or financial relationships that could be construed as a potential conflict of interest.

## Publisher’s note

All claims expressed in this article are solely those of the authors and do not necessarily represent those of their affiliated organizations, or those of the publisher, the editors and the reviewers. Any product that may be evaluated in this article, or claim that may be made by its manufacturer, is not guaranteed or endorsed by the publisher.
